# Experimental study on the impact of water flow velocity on internal erosion of granite residual soil

**DOI:** 10.1038/s41598-025-06012-x

**Published:** 2025-08-04

**Authors:** Shaofeng Wan, Hong Pan, Guanyong Luo, Sige Peng

**Affiliations:** 1 School of Civil Engineering and Transportation, South China University of Technology, Guangzhou, 510640 China; 2 The State Key Laboratory of Subtropical Building Science, South China University of Technology, Guangzhou, 510640 China; 3 School of Civil and Transportation Engineering, Guangdong University of Technology, Guangzhou, 510006 China

**Keywords:** Granite residual soils, Internal erosion, Erosion amount, Wetting front, Erosion channel, Civil engineering, Natural hazards, Hydrology

## Abstract

The internal erosion effect causes fine particles in the soil to move through seepage, and the loss of these fine particles leads to changes in porosity, which in turn affects the soil’s hydraulic properties and mechanical performance, posing a threat to the safety of dam and levee engineering. To understand the formation and development of internal erosion under reverse seepage, a simulation test device for internal erosion was designed, and experiments were conducted on three granite residual soil samples with identical soil properties under different water flow speeds (25 L/H, 50 L/H, and 100 L/H). By comparing and analyzing the wetting front, the amount of internal erosion, and the water content, the influence of water flow speed on reverse seepage internal erosion was studied. The results show that under reverse internal erosion, as the water flow speed increases, the internal erosion rate accelerates, as evidenced by the faster advancement of the wetting front and the increase in cumulative internal erosion. As internal erosion develops, the fine particle accumulation curve enters a stable phase. After the soil’s water content reaches its peak, it slightly decreases and then remains relatively stable. Fluctuations in the soil water content occur due to the formation of preferential internal erosion channels or the redeposition of fine particles. The soil particle movement, fine particle loss, and redeposition caused by internal erosion create an internal erosion channel that narrows from the inlet to the outlet.

## Introduction

Internal erosion is a non-local phenomenon. During the internal erosion process, fine particles are transported through the pores of the solid matrix^1,2^. The loss of these fine particles affects the hydraulic properties and mechanical performance of the soil^3^, and is one of the main causes of failure in dams, levees, and flood embankments. Therefore, the study of internal erosion mechanisms is an important research topic in the fields of geotechnical engineering and hydraulic engineering^4–9^.

The phenomenon of internal erosion has attracted widespread attention from both domestic and international scholars, with a large number of research studies focusing on its development process, mechanism, and the establishment of erosion models. Some researchers have conducted numerical simulations of the formation and development of piping erosion channels in earth-rock dams by artificially altering permeability coefficients or setting boundary conditions for internal erosion based on formulas derived from the critical hydraulic gradient^10^. Jie et al.^11^ developed an elastoplastic constitutive model for sandy soil mixtures to monitor the effect of changes in porosity and fine particle content on the skeleton behavior of the soil caused by internal erosion. Hosn^12^ developed a preliminary numerical extraction program capable of simulating the erosion process by considering the microstructure of granular materials and the hydraulic loading during internal erosion. Meng-Xi et al.^13–15^ used numerical simulations to study particle erosion and transport phenomena in porous media, investigating the formation mechanism and development process of internal erosion. Luo et al.^16^ discussed the development of internal erosion from a seepage-erosion-stress coupling perspective.Lu^17^ and Xi-An^18^ improved the classical seepage erosion calculation formulas, providing strong support for the study of seepage erosion effects. Meng-Xi^19^ and colleagues established a quantitative relationship between model parameters and particle loss, thus enabling a quantitative description of the effect of particle loss on stress–strain behavior. Luc Schol-tès et al.^20^ conducted numerical simulations using two distinct microscopic mechanical models and proposed a multiscale approach to assess the impact of internal erosion on the mechanical properties of granular media.

At the same time, more scholars have conducted indoor model experiments to study the mechanisms and phenomena of internal erosion. Chang et al.^21^ performed laboratory erosion tests under three different stress paths in a complex stress state, aiming to investigate the initiation and development of internal erosion, as well as the effect of stress state on the critical hydraulic gradient. Bendahmane, Moffat, et al. studied the erosion mechanisms of clay and sand under steady seepage conditions. Non-cohesive soils may exhibit internal instability, where fine particles migrate through the voids between coarse particles^22–26^. Li^27^ and Liu^28^ found that soils with internal instability, influenced by seepage flow, are more prone to internal erosion. The initiation of internal instability in soils is controlled by particle gradation. Fannin et al.^22,29,30^ introduced a method to assess soil internal instability based on particle size distribution in laboratory tests. Chen^31–34^ and his team conducted model tests on the development of internal erosion, focusing on two factors: the particle composition of the sample and changes in soil layer structure. To investigate the overall description of the subsurface erosion phenomenon, it is necessary to link the external hydraulic conditions of the specimen with the internal soil changes. Sibille et al.^35^ proposed a power-based constitutive relationship, starting from the material property of erosion resistance, and introduced an energy-based constitutive relation in which the hydraulic load is represented by the accumulated dissipated energy, and the corresponding erosion is represented by the accumulated eroded mass. Inspired by the energy-based constitutive relationship, Kodieh et al.^36^ proposed a new energy-based constitutive relation that better reproduces the evolution of the accumulated erosion mass. Rachel et al.^37^ proposed an energy-based method to describe seepage development, in which this energy method links the accumulated erosion mass of the soil to the accumulated energy dissipated by the hydraulic flow.

However, the aforementioned studies do not explore the effect of water flow velocity on internal erosion. Existing research has shown that water flow velocity is one of the key factors influencing erosion^38^. Therefore, this study utilizes a self-designed soil column internal erosion simulation device, with granite residual soil from the erosion area of the Pearl River Delta as the test material, to explore its internal erosion characteristics under different water flow velocities (25 L/H, 50 L/H, 100 L/H). The experiment measures the mass of fine particles released from the soil column sample due to internal erosion, observes the changes in the wetting front during the internal erosion process, and uses soil moisture sensors to analyze the variation in soil moisture content within the soil column during internal erosion. Through these experiments and statistical analyses, the study examines the effect of water flow velocity on the internal erosion of granite residual soil.

## Internal erosion principle

Internal erosion occurs under the combined action of rainwater and groundwater, making water the decisive factor in the internal erosion process of granite residual soil. Water flows through the pores and cracks within the soil, continuously carrying away fine particles, damaging the soil structure, and increasing the spacing between internal joints and fractures. These fractures eventually connect to form seepage channels, accelerating soil erosion. When internal erosion develops to a certain extent, it may damage roadbeds, cause the collapse of dams, embankments, and flood levees, or even trigger landslides.

Assuming that, based on the flow velocity, a portion of the fine particles can be eroded, transported, and deposited by the flow; in contrast, this effect is ignored for coarse particles, and their density is considered constant over time. This implies that the hydraulic gradient at any point within the soil remains below the critical hydraulic threshold for erosion in granular soils^39^.

Using the mass conservation equation, the fine particles eroded and carried by the flow equal the reduction of fine particles in the soil column.

The cumulative rate ${m}_{a}$ of fine particle mass transported into a unit volume of soil is expressed as:

1 $$m_{a} = - \left( {\frac{{\partial q_{tr,x} }}{\partial x} + \frac{{\partial q_{tr,y} }}{\partial y} + \frac{{\partial q_{tr,z} }}{\partial z}} \right),$$

In the equation, $q_{tr,x} ,{ }q_{tr,y} ,{ }q_{tr,z}$ denotes the area flux.

Therefore, the mass conservation equation for fine particle transport within a unit volume of soil is expressed as:

2 $$\frac{\partial {\rho }_{tr}}{\partial t}=-\left(\frac{\partial {q}_{tr,x}}{\partial x}+\frac{\partial {q}_{tr,y}}{\partial y}+\frac{\partial {q}_{tr,z}}{\partial z}\right)+{q}_{er}-{q}_{dp},$$

In the equation, ${q}_{er}$ denotes the erosion rate of fine particles per unit volume of soil, and ${q}_{dp}$ denotes the deposition rate of fine particles per unit volume.

According to Darcy’s law, $q_{tr,x} ,{ }q_{tr,y} ,{ }q_{tr,z}$ are expressed as:

3 $$\begin{aligned} q_{tr,x} & = \rho_{tr} \cdot v_{x} , \\ q_{tr,y} & = \rho_{tr} \cdot v_{y} , \\ q_{tr,z} & = \rho_{tr} \cdot v_{z} , \\ \end{aligned}$$

In the equation, ${v}_{x}, {v}_{y}, {v}_{z}$ represents the velocity of fine particles transported by water in the X, Y, and Z directions.

It is important to note that Eq. (3) does not imply that the velocity of fine particle transport is the same as the flow velocity of water. In other words, the velocity of the liquid flowing out of the soil column is not the same as the velocity of water entering the soil column. It simply represents the ratio of the liquid volume entering the soil per unit time to the total area perpendicular to the velocity vector. Therefore, ${\rho }_{tr}$ refers to the ratio of the mass of fine particles transported per unit volume of soil to the volume of liquid entering the same unit volume of soil in the same time period.

Substituting Eq. (3) into Eq. (2), the continuity equation for fine particle transport is obtained:

4 $$\frac{\partial \left({\rho }_{tr}{v}_{x}\right)}{\partial x}+\frac{\partial \left({\rho }_{tr}{v}_{y}\right)}{\partial y}+\frac{\partial \left({\rho }_{tr}{v}_{z}\right)}{\partial z}+\frac{\partial {\rho }_{tr}}{\partial t}={q}_{er}-{q}_{dp},$$

The seepage velocity depends on the hydraulic gradient h in Darcy’s law:

Multiply both sides of Eq. (4) by a weighting function $\rho_{{{\text{tr}}}}^{*}$, and integrate over an arbitrary volume $V^{*}$ to get:

5 $$\begin{aligned} & \int\limits_{{V^{*} }} {\rho_{{{\text{tr}}}}^{*} \left( {\frac{{\partial v_{x} }}{\partial x} + \frac{{\partial v_{y} }}{\partial y} + \frac{{\partial v_{z} }}{\partial z}} \right)\rho_{tr} dV} + \int\limits_{{V^{*} }} {\rho_{{{\text{tr}}}}^{*} \left( {V_{x} \frac{{\partial \rho_{tx} }}{\partial x} + V_{y} \frac{{\partial \rho_{ty} }}{\partial y} + V_{z} \frac{{\partial \rho_{tr} }}{\partial z}} \right)dV} \\ & \quad + \int\limits_{{V^{*} }} {\rho_{{{\text{tr}}}}^{*} \frac{{\partial \rho_{tr} }}{\partial t}dV = } \int\limits_{{V^{*} }} {\rho_{{{\text{tr}}}}^{*} \left( {q_{er} - q_{dp} } \right)dV} , \\ \end{aligned}$$

Since ${\rho }_{\text{tr}}^{*}$ and ${V}^{*}$ can be arbitrarily chosen, Eq. (5) is equivalent to the continuity Eq. (4) for an infinitesimal volume.

Assume that the distribution of the hydraulic head $h\left( {x,y,z,t} \right)$ and the density $\rho_{tr} \left( {x,y,z,t} \right)$ of transported fine particles in the soil depends on the node values in vectors $h\left( t \right)$ and $\rho_{tr} \left( t \right)$, and is expressed through the interpolation functions $f_{h} \left( {x,y,z} \right)$ and $f_{r} \left( {x,y,z} \right)$, then:

6a $$h\left( {x,y,z,t} \right) = f_{h}^{T} \left( {x,y,z} \right) \cdot h\left( t \right) = h^{T} \cdot f_{h} ,$$

6b $$\rho_{tr} \left( {x,y,z,t} \right) = f_{p}^{T} \left( {x,y,z} \right) \cdot \rho_{tr} \left( t \right) = \rho_{tr}^{T} \cdot f_{p} ,$$

During the internal erosion process, the concentration of fine particles ${\rho }_{f}$ in the soil sample decreases over time t, from an initial value ${\rho }_{{f}_{0}}\left(t=0\right)$ to a long-term value ${\rho }_{{f}_{\infty }}$. In Sterpi’s soil column experiments, it was observed that under prolonged internal erosion, the density of fine particles increased with the hydraulic gradient^40^, which, from a physical perspective, means it increases with seepage velocity v. However, the ratio of the increase in density $\Delta \left({\rho }_{{f}_{0}}-{\rho }_{{f}_{\infty }}\right)$ due to long-term erosion to the corresponding increase in seepage velocity $\Delta v$ decreases as v increases, and the value of $\Delta \left({\rho }_{{f}_{0}}-{\rho }_{{f}_{\infty }}\right)/\Delta v$ tends to decrease with increasing v. Therefore, it can be assumed that the length density ${\rho }_{{f}_{\infty }}$ is a decaying function of v, approaching 0 as the flow velocity increases. The relationship between them can be expressed as:

7a $$\rho_{{f_{\infty } }} \left( v \right) = \rho_{{f_{0} }} - \left( {\rho_{{f_{0} }} - \rho_{{f_{\infty } }}^{*} } \right) \cdot \frac{v}{{v^{*} }}0 \le v\left( t \right) \le v^{*} ,$$

7b $$\rho_{{f_{\infty } }} \left( v \right) = \rho_{{f_{\infty } }}^{*} - \alpha \cdot \log \left( {v/v^{*} } \right){ }v^{*} \le v\left( t \right),$$

7c $$\rho_{{f_{\infty } }} \ge 0,$$

In the equation, $v^{*}$ is the minimum flow velocity required to transport fine particles in the soil, $\rho_{{f_{\infty } }}^{*}$ is the corresponding long-term density (the minimum density of fine particles in the soil), $\rho_{{f_{0} }}$ is the initial density of fine particles (at t = 0), and α is the model parameter.

The internal erosion rate ${q}_{er}$ depends on the current flow velocity v and the concentration of fine particles ${\rho }_{f}\left(t\right)-{\rho }_{{f}_{\infty }}$ in the soil at the current state, and is expressed as:

8 $$q_{er} \left( {t,v} \right) = \beta \cdot v \cdot \left[ {\rho_{f} \left( t \right) - \rho_{{f_{\infty } }} \left( v \right)} \right],$$

In the equation, β is a model parameter determined by fitting experimental results. Meanwhile, based on experimental results of seepage-induced internal erosion, Sterpi derived a model for fine particle erosion and transport. Specifically, when the flow path for fine particle transport in the soil column significantly exceeds the average particle size of the soil sample, redeposition and pore clogging of the eroded fine particles should be considered. This suggests that internal erosion involves the effective separation and transport of particles, requiring separate analysis of fine particle separation and transport. The specific relationship is expressed as:

9 $${\mu }_{e}={\mu }_{0}\left[1-\mathit{exp}\left(-{\left(\frac{t}{{t}_{0}}\right)}^{b}\cdot \frac{{i}^{c}}{a}\right)\right],$$

In the equation, ${\mu }_{e}$ is the weight percentage of eroded fine particles, i is the hydraulic gradient, t is time, ${\mu }_{0}$ is the initial concentration of fine particles, and a, b, and c are dimensionless parameters.

## Test device and materials

### Test device

**Fig. 1:**
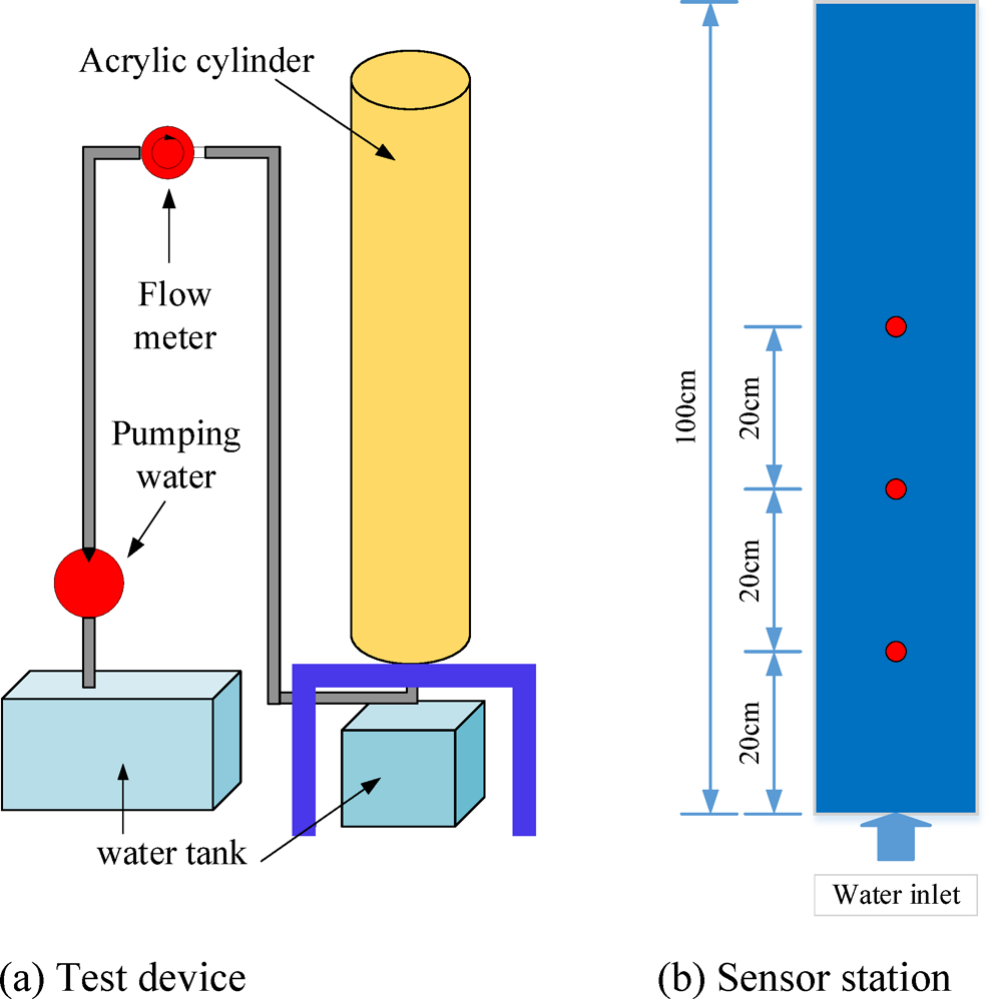
Test device.

To further investigate the internal erosion of granite residual soil under water seepage, an indoor soil column erosion experimental setup was designed. This setup effectively simulates the internal erosion process and monitors key parameters. The experimental device, shown in Fig. 1a, mainly consists of components such as an acrylic cylinder, a water pump, a flowmeter, a filter, soil moisture sensors, and a metal mesh.

The core component of the device is a transparent acrylic cylinder with an inner diameter of 20 cm and a length of 100 cm. The cylinder offers excellent visibility, making it easy to observe the experimental process and record data. A transparent scale is attached to the outer wall of the acrylic cylinder, allowing for real-time tracking of changes in the wetting front inside the soil column over time.

On the left side of the device, a transparent acrylic water tank is installed to provide a stable water source. The water tank is connected to the acrylic cylinder via a connecting pipe, with a water pump and flowmeter installed on the pipe to control the flow rate and accurately measure the flow. To ensure uniform distribution of water entering the soil column, a filter is specifically designed and installed at the inlet of the acrylic cylinder to eliminate experimental errors caused by uneven local flow rates. Additionally, to prevent particles flowing out downstream from entering the recirculation system, a metal mesh with a pore size of 3 mm is installed downstream of the cylinder.

To obtain real-time data on changes in soil moisture within the soil column, soil moisture sensors and pore pressure sensors were embedded at three different positions inside the acrylic cylinder, as shown in Fig. 1b. These sensors are located 20 cm, 40 cm, and 60 cm from the inlet and are used to dynamically monitor changes in soil moisture content and pore pressure at different positions. This ensures a comprehensive reflection of the wetting front progression and the spatial distribution of moisture within the soil.

A transparent water tank is placed below the acrylic cylinder to collect the water that permeates out and the fine particles carried away during the internal erosion process. By observing and measuring the particle deposition in the water tank, the intensity of the internal erosion phenomenon and the patterns of particle migration can be quantitatively analyzed. Additionally, the transparent design of the water tank allows for easy observation of the effects of the seepage process on water quality.

### Testing material

**Fig. 2:**
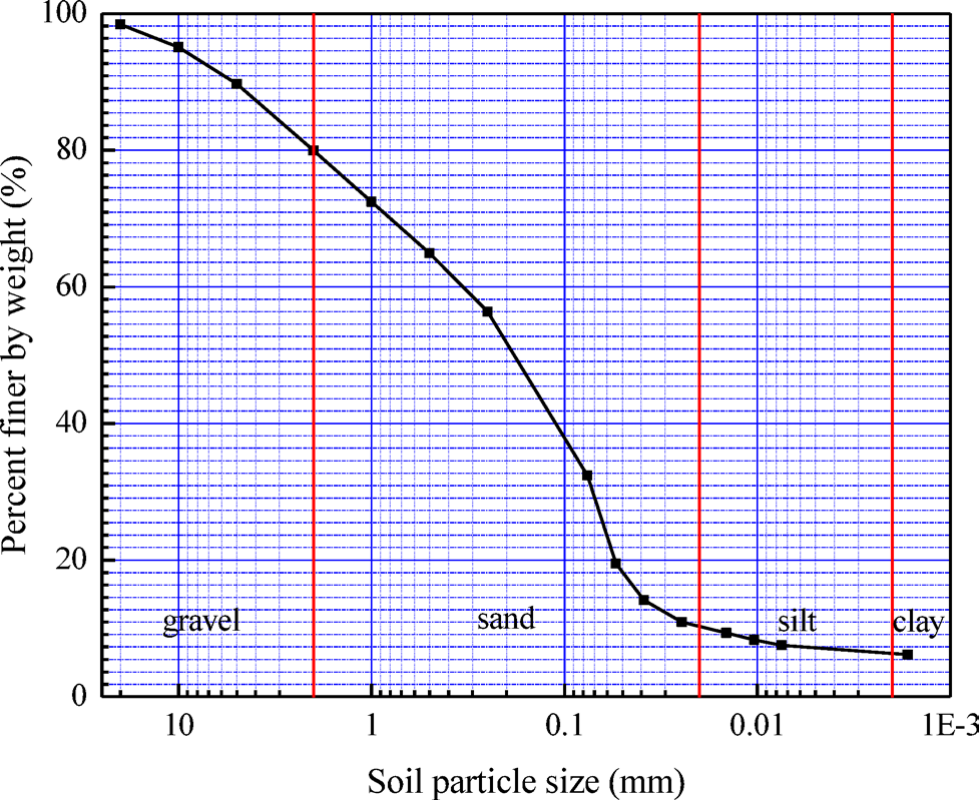
Grain size distribution of granite residual soil.

Granite residual soils are widely distributed in the Pearl River Delta region. These soils have a relatively loose structure with weak particle bonding. Due to their loose structure, these soils exhibit high permeability, which can easily lead to problems such as soil erosion and foundation settlement. The material used for the soil column internal erosion simulation experiment was obtained from granite residual soils in the Pearl River Delta region. A particle size distribution test was performed on the material, and the particle size distribution curve is shown in Fig. 2. It can be observed that particles smaller than 0.075 mm account for approximately 32% of the total. The natural moisture content of the soil sample is 25.75%, its natural dry density is 1.50 g/cm^3^, and the specific gravity of the soil is 2.734.

### Test procedure

To investigate the effect of flow velocity on the internal erosion behavior of granite residual soils, this study used the experimental setup shown in Fig. 1 to conduct internal erosion simulation experiments at different flow velocities (25 L/H, 50 L/H, 100 L/H). The experiment was divided into three stages: soil sample preparation, filling, and the experimental process. The specific procedures are as follows:

The first step is to prepare the soil samples. Larger stones are removed from the granite residual soil sample to reduce the impact of heterogeneous particles on the experimental results. The treated soil is then spread out and air-dried for 2–3 days until it reaches a naturally dried state. Afterward, an appropriate amount of water is added to achieve the target moisture content of 10%, and the mixture is evenly stirred and left to stand for 2 days. To prevent moisture evaporation, the soil sample is covered with plastic film during this period to ensure that the moisture is thoroughly mixed with the soil particles. Once the standing period is complete, the soil sample is divided into several portions for later use.

**Fig. 3:**
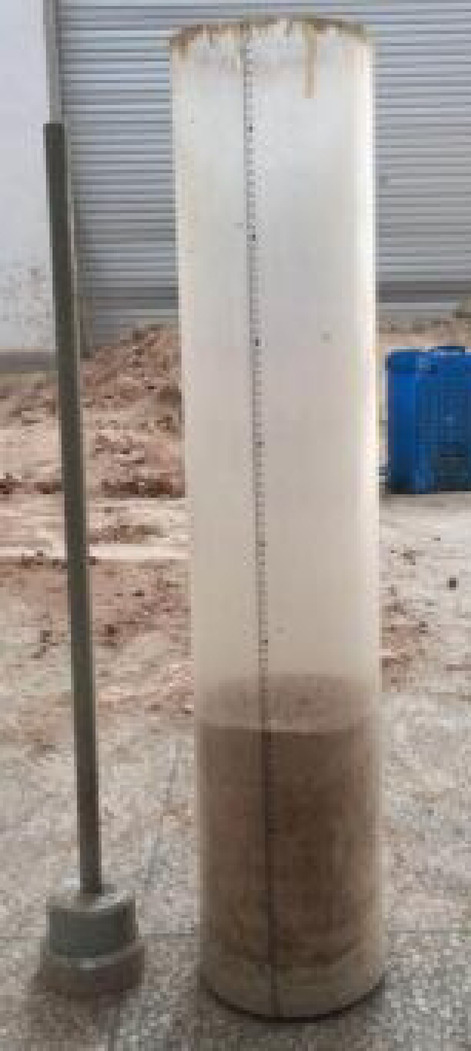
Soil column filling.

**Fig. 4:**
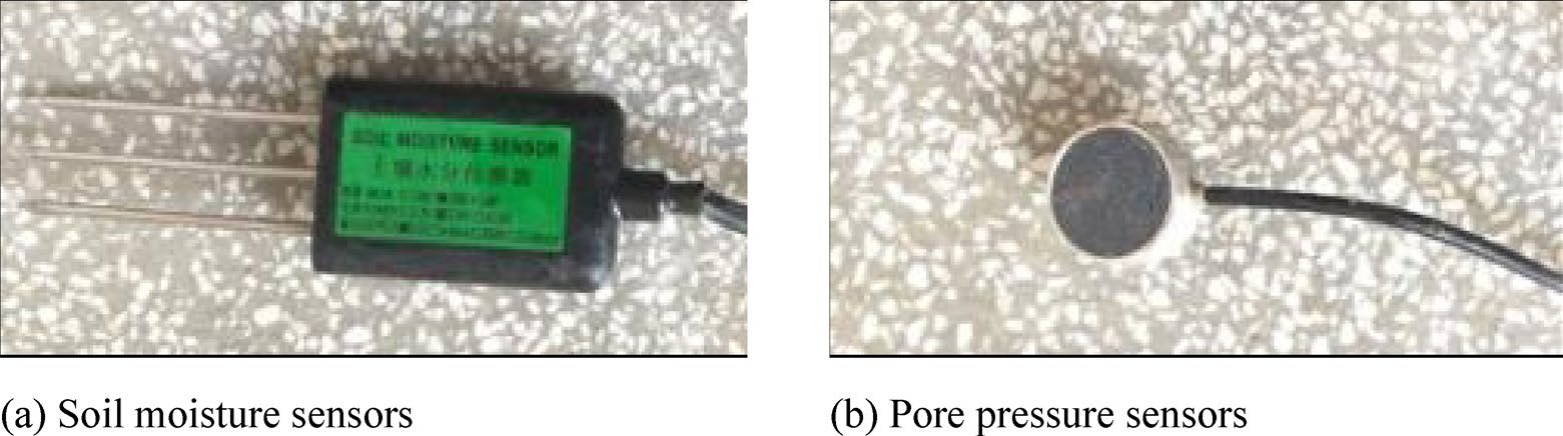
Sensor station.

The prepared soil is layered and placed into the acrylic cylinder, with each layer having a height of 10 cm. To ensure the integrity of the soil column after filling, the surface of each layer is scarified to enhance the interlayer bonding. After each layer is added, a specialized tool is used to compact it (as shown in Fig. 3) to ensure uniform density. This process is repeated until the height of the soil column reaches the design requirement of 100 cm. At the 20 cm, 40 cm, and 60 cm heights, soil moisture sensors and pore pressure sensors are embedded (as shown in Fig. 4). These sensors are used to record changes in soil moisture content and pore water pressure at different depths within the soil column in real time.

After filling the soil sample, the acrylic cylinder is vertically fixed onto the experimental setup. Water is injected from the lower end of the setup, flowing through the filter and entering the soil column at a constant flow rate (25 L/H, 50 L/H, 100 L/H; L/H means Liter/Hour). The water flows upward and infiltrates the soil. During the experiment, the rise of the wetting front in the soil column is observed in real-time through the transparent scale on the outer wall of the acrylic cylinder. Meanwhile, soil moisture sensors record the changes in moisture content at 20 cm, 40 cm, and 60 cm depths over time. To study the loss of fine particles, the effluent from the outlet at the other end of the soil column is collected at fixed time intervals (every 10 min). The collected liquid is allowed to settle, and the precipitated particles are then dried in an oven and weighed. By recording the changes in the weight of fine particles at different time points, the cumulative loss of fine particles over time is obtained.

Through the analysis of experimental data, the influence of flow velocity on the internal erosion characteristics of soil can be studied, including the cumulative loss of fine particles, the advancement rate of the wetting front, and the spatial distribution characteristics of soil moisture. Meanwhile, the pore water pressure data recorded by the sensors further reveal the impact of flow velocity on the hydraulic properties of the soil.

## Internal erosion process

**Fig. 5:**
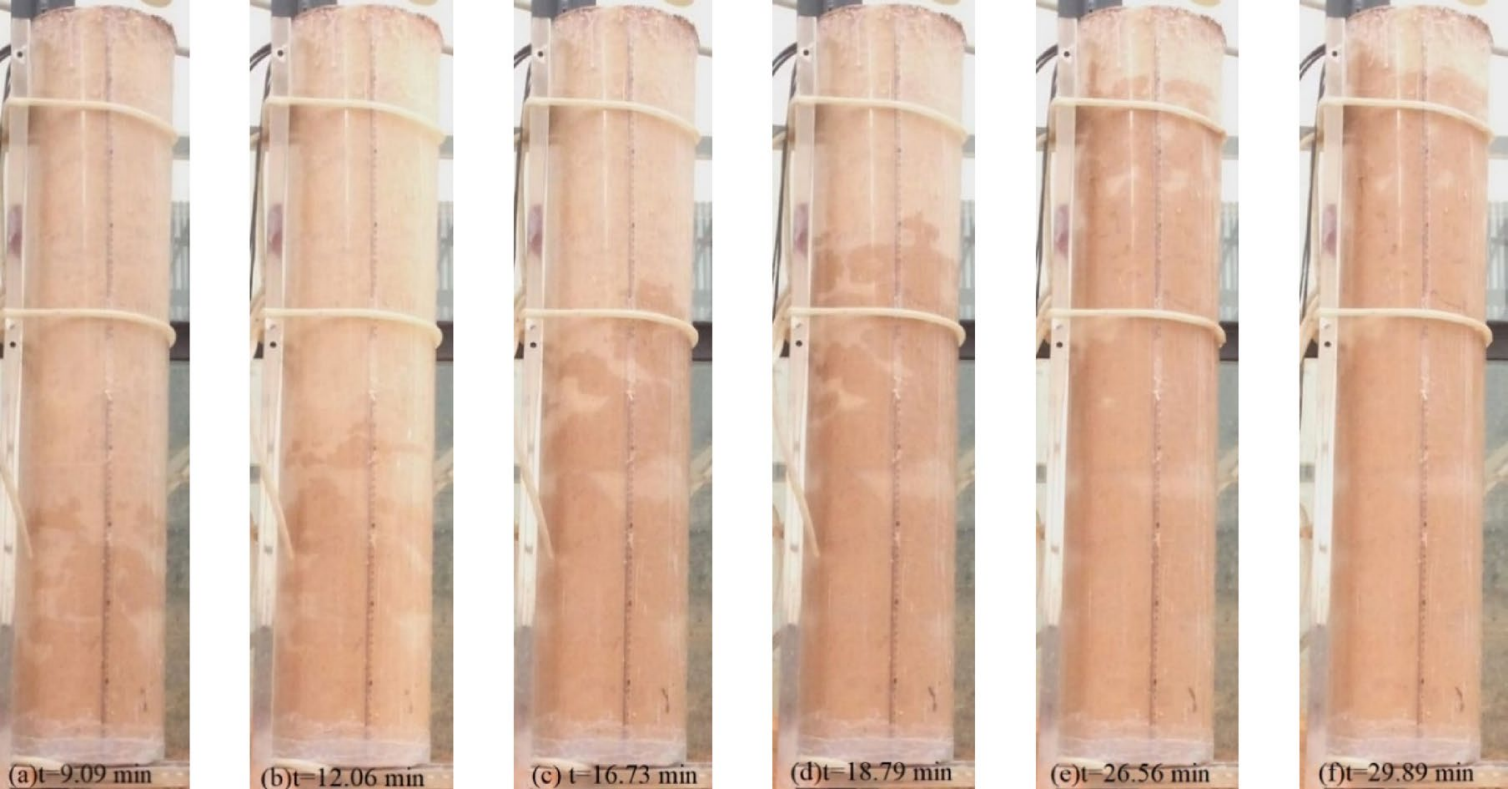
Wetting front propulsion when the flow rate is 25 L/H.

During the experiment, water flows into the vertically placed soil column at different speeds. Fine particles in the upstream section of the soil column are gradually eroded by the water seepage force, leading to internal erosion within the column. As the water infiltrates the soil column, the wetting front continuously advances. At a flow velocity of 25 L/H, the advancement of the wetting front during the internal erosion process is shown in Fig. 5. At t = 9.09 min (9 min and 5.4 s), the wetting front reaches 30 cm; at t = 12.06 min, it reaches 40 cm; at t = 16.73 min, it reaches 60 cm; at t = 18.79 min, it reaches 70 cm; at t = 26.56 min, it reaches 90 cm; and at t = 29.89 min, it reaches 100 cm.

**Fig. 6:**
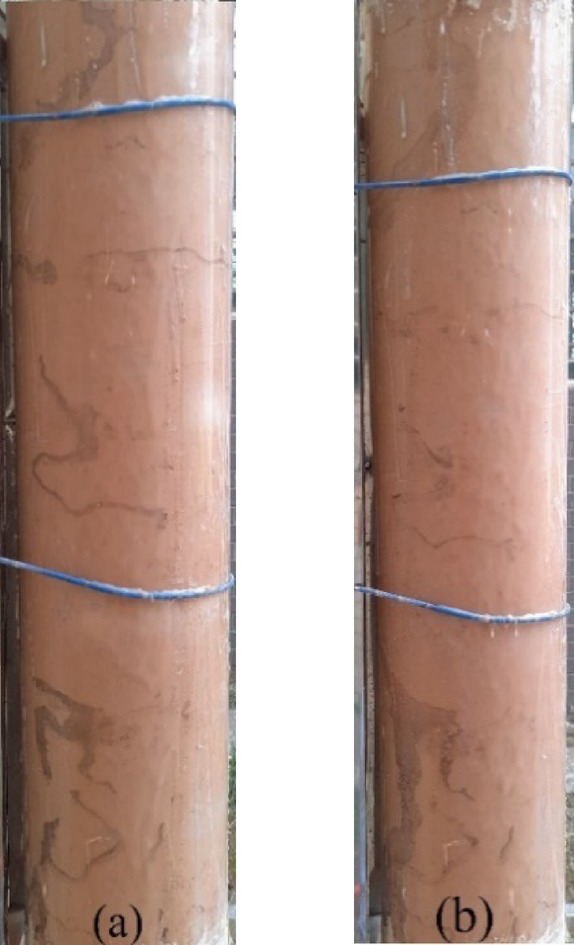
Development process of internal erosion channel when flow rate is 100 L/H.

When observing the internal erosion phenomenon in the soil column, it can be noted that as fine particles migrate, large preferential internal erosion channels are formed within the soil column, leading to localized voiding^41^. For instance, under a flow velocity of 100 L/H, a more significant preferential internal erosion channel is formed during the internal erosion process, as shown in Fig. 6. The permeability coefficient of the soil sample changes, resulting in a sudden increase in the turbidity of the effluent. At the same time, fine particles are transported by the water flow to the water tank on the right. As time progresses, the fine particle content in the effluent fluctuates, showing an increase, followed by a gradual decrease. This is reflected in the water becoming turbid and then gradually clearing. The fluctuation in the amount of internal erosion of fine particles is significant, with clean water observed at times and turbid water at others during the internal erosion process. This indicates that the development of internal erosion channels is a complex and repetitive process.

At the beginning of the internal erosion process, air bubbles move inside the soil column, and soil fine particles are displaced from the soil framework particles by the infiltrating water. The fine particles roll under the influence of the seepage force, as shown in Fig. 6a. These fine particles gradually flow out of the internal erosion channels as the seepage water moves through. After some time, internal erosion forms erosion channels within the soil column. As time progresses, internal erosion continues. As shown in Fig. 6b, the front end of the internal erosion channel expands further, but with increasing internal erosion distance, the size of the erosion channel begins to decrease. This suggests that the front part of the internal erosion channel is primarily affected by internal erosion, while the rear part is mainly influenced by transport and redeposition processes.

## Test result analysis

### Wetting front

**Fig. 7:**
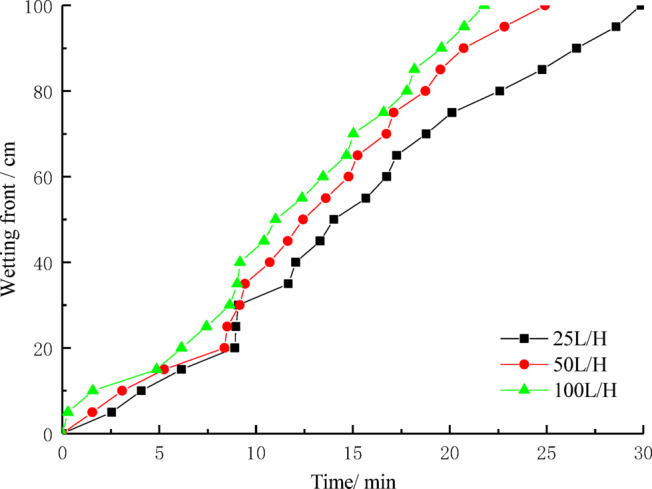
The relationship between wetting front and time.

The analysis results of the influence of different flow rates on the wetting front are shown in Fig. 7. As the flow rate increases, the speed of wetting front advancement also increases, reflecting a positive correlation between flow rate and the advancement of the wetting front. Specifically, when the flow rate is 25 L/H, it takes 29.89 min for the wetting front to reach 100 cm. When the flow rate increases to 50 L/H, the time required for the wetting front to reach 100 cm decreases to 24.93 min. When the flow rate is further increased to 100 L/H, the time for the wetting front to reach 100 cm drops to 21.78 min. This result indicates that the flow rate largely determines the speed at which the wetting front advances. Under higher flow rates, water is able to transfer more quickly into the deeper layers of soil, promoting the forward movement of the wetting front.

Although the speed of the wetting front increases with flow rate, the relationship between flow rate and wetting front movement is not a simple linear one. Within the first 20 cm of the soil column, the advancement of the wetting front is nearly linear, with a speed of approximately 2.24–3.25 cm/min. However, between 20 and 60 cm, there is a significant jump in the advancement speed, with the speed increasing sharply to 5.10–5.47 cm/min. This change may be related to the formation of preferential internal erosion channels within the soil column. These internal erosion channels are typically formed by water flow creating rapid pathways in the soil, especially when larger particles or larger pores are present. As water moves through the soil, it may concentrate along these voids or weak points, gradually forming one or more rapid channels. These channels can bypass the surrounding soil structure and directly transport water to deeper layers. At this stage, water migration is no longer a uniform diffusion process but is dominated by rapid infiltration through these preferential channels. Initially, the wetting front advances more slowly, while water moves farther through the preferential internal erosion channels. As the water gradually infiltrates the soil column, the speed of the wetting front eventually stabilizes.

### Internal erosion amount

**Fig. 8:**
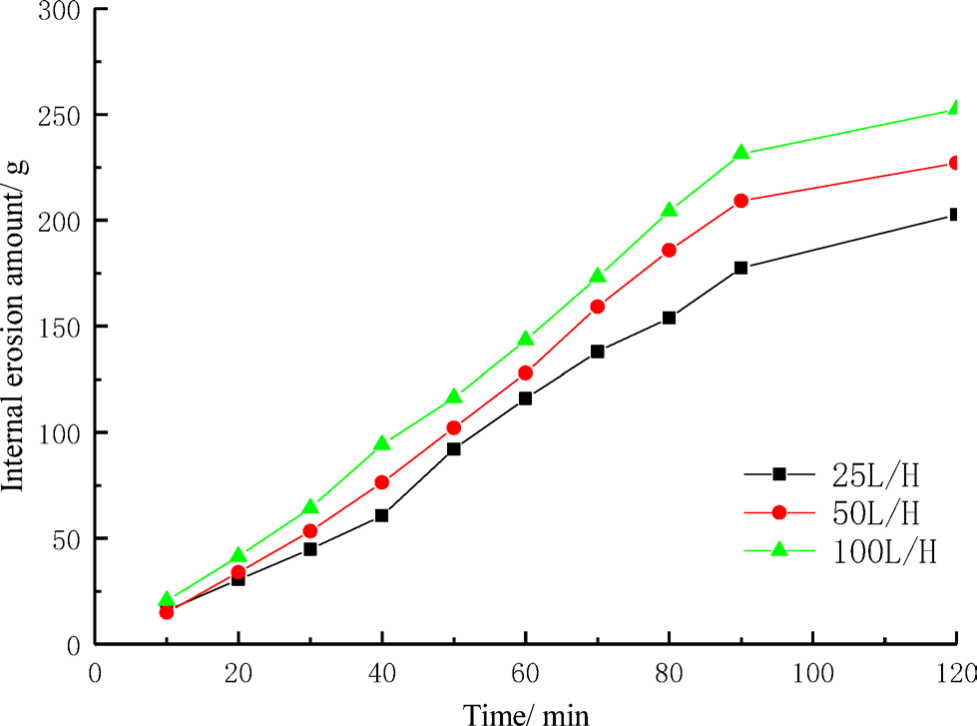
The relationship between internal erosion amount and time.

The experiment simulated internal erosion under different flow rates and measured and analyzed the mass of fine particles lost (internal erosion) over a specific period of time. After collecting liquid samples from each experiment, they were allowed to settle, then dried and weighed to determine the mass of fine particles lost due to internal erosion. The results of the analysis of the impact of different flow rates on internal erosion in the soil are shown in Fig. 8. When the flow rate was 25 L/H, the accumulated mass of fine particles in the glass container increased by 202.61 g from 0 to 90 min. When the flow rate increased from 25 to 50 L/H, the accumulated mass of fine particles in the glass container increased from 0 to 226.99 g at 90 min. At a flow rate of 100 L/H, the accumulated fine particle mass reached 252.33 g in 90 min. After 90 min of the experiment, the increase in fine particle accumulation at different flow rates was minimal. This indicates that flow rate has a certain impact on internal erosion. Higher flow rates are more effective at breaking the bond between soil particles, thereby promoting greater fine particle loss.

The faster the water flow, the stronger the water’s impact force and shear force. This enhanced mechanical effect increases the separation and loss of soil particles, leading to a rise in internal erosion. Especially in the initial stage, the loss of fine particles shows a noticeable upward trend because the scouring and pushing action of the water on the soil is stronger, and the loosening and loss of fine particles occur more rapidly. However, after 90 min of the experiment, although the difference in water flow speed still exists, the cumulative amount of fine particles begins to stabilize. This indicates that, over a longer period, the impact force of the water flow gradually weakens, and the loosening and migration of soil particles become limited. This phenomenon may be related to the gradual infiltration of water, which fills the voids in the soil, causing the loss rate of fine particles to decrease.

Under different water flow speeds, the experiment commonly exhibited the phenomenon of preferential internal erosion channels. This phenomenon suggests that under the influence of water flow, certain areas inside the soil column, due to the loosening of particles and the impact of the water flow, are prone to forming efficient internal erosion channels. These channels allow water to pass through at a higher speed, which intensifies the loss of fine particles in that area. This results in a concentrated loss of fine particles, leading to a rapid increase in the cumulative amount of fine particles during certain periods. Differences in the internal structure and pore distribution of the soil column may also cause variations in the internal erosion intensity in different areas, leading to fluctuations in the loss of fine particles.

### Moisture content

**Fig. 9:**
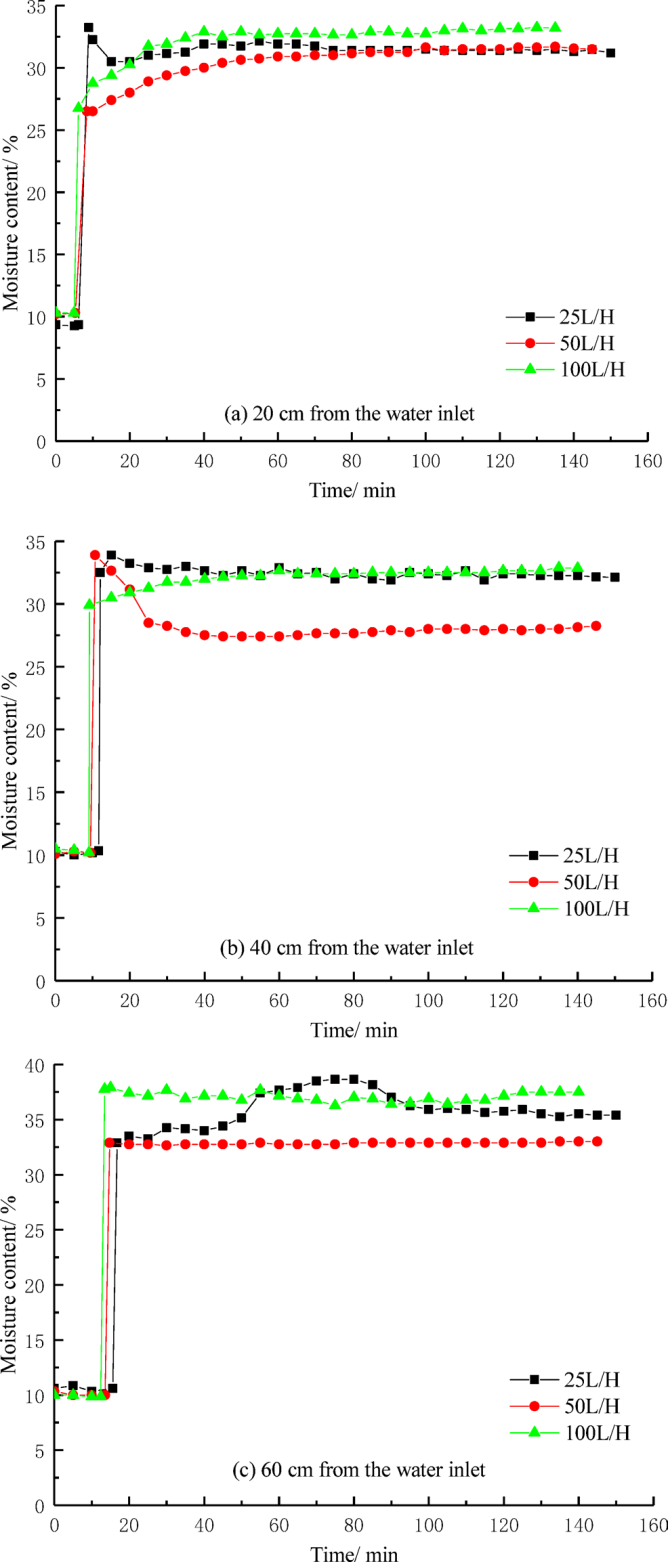
The relationship between moisture content and time.

Figure 9 shows the effect of water flow speed on the soil moisture content at each measurement point. In Fig. 9a, when the water flow speeds are 25 L/H, 50 L/H, and 100 L/H, the soil moisture content at the 20 cm depth of the soil column at the time points of 8.91 min, 8.38 min, and 6.16 min, respectively, suddenly increases to around 35%, and the moisture content remains relatively stable at this level after reaching approximately 35%. It is evident that as the water flow speed increases, the time for internal erosion to occur at the same position in the soil column gradually shortens. With higher water flow speeds, the interaction between the water flow and the soil particles is intensified, leading to faster infiltration of moisture into the interior of the soil column.

In the later stages of internal erosion, the soil moisture content showed fluctuations. These fluctuations were primarily due to the possible formation of preferential internal erosion channels during the erosion process, which influenced the flow path of the water, causing a redistribution of moisture. The formation of preferential channels could cause the water to flow along a specific path, leading to a local increase or decrease in the water flow speed, thereby affecting the measurement of soil moisture content. Especially near the sensors, the moisture in the soil could be redistributed, resulting in sudden increases or decreases in water content. Water flow not only carries moisture but also transports fine soil particles, which may be redeposited in other parts or on the surface of the soil column, creating the so-called “redeposition” phenomenon. The deposition of these fine particles may alter the local soil structure, affecting the moisture conduction path and causing fluctuations in the measured moisture content.

### Pore water pressure

**Fig. 10:**
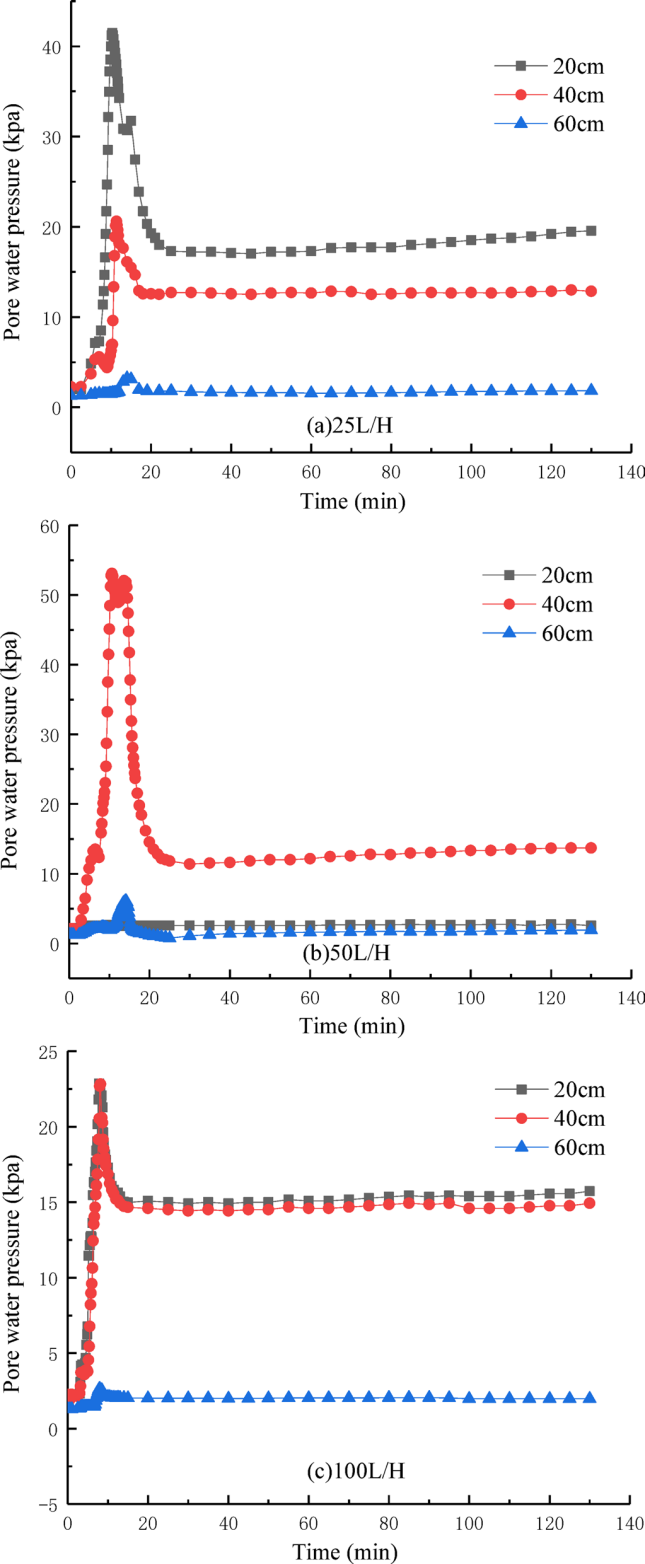
The relationship between pore water pressure and time.

Figure 10 shows the effect of flow velocity on pore pressure at each measurement point. When the flow velocity is 25 L/H, the changes in pore pressure at different depths (20 cm, 40 cm, 60 cm) of the soil column are as follows: at 20 cm, the pore pressure reaches its maximum value of 41.47 kPa at 10.3 min and then stabilizes around 19.5 kPa; at 40 cm, it reaches 20.60 kPa at 11.3 min and then stabilizes around 12.8 kPa; at 60 cm, it reaches 3.25 kPa at 14 min and then stabilizes around 1.83 kPa. The overall trend is clear: before the water flow reaches the point, the pore pressure is nearly zero, and after the flow passes, the pore pressure rises rapidly and stabilizes at a relatively balanced value. At flow velocities of 25 L/H, 50 L/H, and 100 L/H, the stable pore pressures at 60 cm are 1.83 kPa, 1.93 kPa, and 1.98 kPa, respectively.

In Fig. 10b and c, the changes in pore pressure at 20 cm, 40 cm, and 60 cm of the soil column are not exactly the same as in Fig. 10a. In Fig. 10b, the pore pressure at 20 cm remains unchanged because a preferential internal erosion channel formed at this section, causing the water flow to bypass the sensor. In Fig. 10c, the pore pressure extreme at 20 cm is similar to that at 40 cm, also due to the formation of a preferential internal erosion channel, which caused this phenomenon.

## Discussion

The variability and stability of internal erosion are important phenomena observed in the experiment. Although the loss of fine particles increases with the flow velocity in the early stages of the experiment, over time, internal erosion gradually stabilizes. This process reflects the regularity and localized changes in particle loss within the soil column under prolonged internal erosion.

In the early stages of internal erosion, the concentration of particles in the water flow is relatively high due to the loss of fine particles from the soil. Over time, the water flow begins to clear, and the concentration of fine particles gradually decreases. This may be related to the distribution of particles in the soil and the percolation effect of the water flow, which leads to a gradual reduction in the loss of fine particles and a stabilization of internal erosion. As internal erosion continues, the interaction between the water flow and soil particles becomes more complex. Some fine particles may be redeposited in the lower part of the soil column or trapped within the soil, and this redistribution effect further influences the stability of internal erosion. Preferential internal erosion channels and the clogging of soil pores are significant factors in the internal erosion process. They not only affect the loss of fine particles but also contribute to the variability of internal erosion.

Flow velocity is a key factor influencing internal erosion in granite residual soils. It directly determines the advancement speed of the wetting front, the rate of fine particle loss, and the formation characteristics of internal erosion channels. This study not only reveals the effects of different flow velocities on internal erosion but also provides experimental evidence and theoretical support for the prevention and control of internal erosion and the design of soil stability. Future research could further explore the synergistic effects of soil particle gradation and soil structures with different specific gravities on internal erosion behavior.

## Conclusions

This study conducted internal erosion simulation experiments on granite residual soil columns using a self-designed soil column internal erosion simulation device, controlling the flow velocity. Based on the experimental results, the study evaluates the impact of flow velocity on the initiation and development of internal erosion:

1. As the flow velocity increases from 25 to 50 L/H and 100 L/H, the advancement speed of the wetting front accelerates, and internal erosion within the soil column occurs more rapidly. This is reflected in the shortened time required for the wetting front to reach 100 cm, which is 29.89 min, 24.93 min, and 21.78 min, respectively. At the same time, higher flow velocities exacerbate the loss of fine particles and the accumulation of internal erosion within the soil column, with the cumulative loss of fine particles over 90 min being 202.61 g, 226.99 g, and 252.33 g, respectively.
2. The cumulative curve of fine particle loss initially shows a rapid increase, followed by gradual stabilization. In the later stages of the experiment, the infiltrating water under different flow velocity conditions gradually becomes clearer, indicating a weakening of internal erosion. The formation of preferential internal erosion channels and clogging phenomena are particularly significant in the later stages of internal erosion, as reflected in the fluctuations in particle concentration in the effluent.
3. During the internal erosion process, the time at which the soil moisture content reaches its peak shortens as the flow velocity increases, and the peak moisture content tends to stabilize. However, in the later stages of internal erosion, due to the formation of preferential internal erosion channels and the redeposition of fine particles, the soil moisture content fluctuates. This indicates that the development of internal erosion channels is a complex and dynamic process, influenced by the migration and deposition of soil fine particles.
4. The internal erosion process gradually forms an erosion channel within the soil column, which narrows from the water entry end to the water exit end. Its formation and development are influenced by the combined effects of flow velocity, particle gradation of fine particles, and redeposition behavior. Under rapid infiltration conditions, the erosion at the front end of the internal erosion channel is more pronounced, while fine particles at the rear end are more likely to undergo redeposition.

## Data Availability

The datasets used and/or analysed during the current study available from the corresponding author on reasonable request.
